# miR-483-3p, Mediated by KLF9, Functions as Tumor Suppressor in Testicular Seminoma *via* Targeting MMP9

**DOI:** 10.3389/fonc.2020.596574

**Published:** 2021-02-15

**Authors:** Lei Zhang, Yashi Ruan, Zhiqiang Qin, Xian Gao, Kai Xu, Xiaokai Shi, Shenglin Gao, Shouyong Liu, Kai Zhu, Wei Wang, Li Zuo, Lifeng Zhang, Wei Zhang

**Affiliations:** ^1^ Department of Urology, The Affiliated Changzhou No. 2 People’s Hospital of Nanjing Medical University, Changzhou, China; ^2^ Graduate School of Nanjing Medical University, Nanjing, China; ^3^ Department of Urology, The First Affiliated Hospital of Nanjing Medical University, Nanjing, China; ^4^ Department of Urology, Taizhou People’s Hospital, The Fifth Affiliated Hospital of Medical School of Nantong University, Taizhou, China; ^5^ Department of Urology and Transplantation, Nanjing First Hospital, Nanjing Medical University, Nanjing, China; ^6^ Department of Oncology, The First Affiliated Hospital of Nanjing Medical University, Nanjing, China

**Keywords:** miR-483-3p, testicular germ cell tumor, seminoma, MMP9, KLF9

## Abstract

**Background:**

Seminoma (SEM) is the most frequent testicular germ cell tumor with a high incidence in young men. The present study aims to explore the function and regulatory mechanism of miR-483-3p in SEM.

**Methods:**

RT-qPCR was performed to investigate miR-483-3p levels in SEM tissues. The effect of miR-483-3p on TCam-2 cells was assessed by CCK-8, colony formation, cell migration, and invasion assays. Luciferase reporter assays were performed to investigate the interaction between miR-483-3p and MMP9, and then the recovery experiments were performed. Moreover, the potential upstream regulator of miR-483-3p was predicted based on JASPAR database.

**Results:**

miR-483-3p was down-regulated in SEM tissues versus paracancerous normal tissues. The expression level of miR-483-3p was significantly associated with tumor stage by RT-qPCR. Functionally, miR-483-3p over-expression suppressed cell growth, migration, and invasion in SEM cell lines. Mechanically, miR-483-3p negatively regulated MMP9 by directly binding to its 3′-UTR. The over-expression of miR-483-3p could reverse the promoting role of MMP9 over-expression on the proliferation, migration, and invasion of TCam-2 cells. Moreover, KLF9 was identified as a potential upstream regulator of miR-483-3p and functions as a tumor suppressor.

**Conclusions:**

In general, our study suggested that miR-483-3p could inhibit the cell growth, migration, and invasion of testicular SEM by targeting MMP9. Moreover, KLF9 is an upstream positive regulator of miR-483-3p and also functions as a tumor suppressor in SEM.

## Introduction

Testicular germ cell tumor (TGCT) is the most common solid malignancy in young men with the incidence rising constantly over the past decades (1, 2). TGCT is usually stratified into two histologic groups: seminoma (SEM) and non-seminomas (NSEM), while the latter can be further divided into embryonal carcinoma (EC), yolk sac tumors, teratoma, and choriocarcinoma (3, 4). SEM develops from premalignant intratubular germ cell neoplasia and shares a similar gene expression and epigenetic profile with primordial germ cells (5). The majority (~70%) of SEM patients present with clinical stage I disease and have a very good overall survival after surgical treatment and radiotherapy (6). However, the metastatic and relapse rates of high-grade SEM are high, and as a result, positive clinical surveillance is essential (7). Higher-dose radiotherapy and platin-based chemotherapy are recommended for Stage II/III SEM with lymph nodes and distant metastasis, which reveals high risks and unfavorable prognosis (6). Therefore, it is imperative to further illuminate the molecular mechanisms involved in the progression of SEM and provide potential biomarkers for the clinical treatment, surveillance, and prognosis of patients with SEM.

MicroRNAs (miRNAs) are a class of endogenous small non-coding RNA molecules containing 18–25 nucleotides, which always function as post-transcriptional regulators of the mRNAs expression through binding to the 3′-untranslated region (3′-UTR) of their targets (8, 9). miRNAs have been found to be involved in a variety of biological processes, including tumor occurrence and progression (10, 11). Growing evidence has indicated the aberrant expression of miRNAs significantly associated with the development of testicular SEM (12–15). Recent study has found that miR-483-3p could suppress the cell proliferation and promote cell apoptosis in breast cancer by directly regulating SOX3 (16). Moreover, epigenetic silencing of miR-483-3p could promote the resistance of gefitinib in non-small-cell lung cancer (NSCLC) (17). However, the role of miRNA-483-3p in testicular SEM remains unclear and to be explored.

In the current study, we identified miR-483-3p as a tumor suppressor in human testicular SEM. miR-483-3p could inhibit the cell growth, migration and invasion of tumor cells by directly targeting matrix metalloproteinase 9 (MMP9). Moreover, the transcriptional factor (TF) KLF9 is found to be the upstream regulatory factor of miR-483-3p. The novel pathway KLF9/miR-483-3p/MMP9 might contribute to profounder understanding of the genesis and the development of human testicular SEM.

## Materials and Methods

### Clinical Samples

Twenty pairs of testicular SEM tissues and paracancerous normal tissues were collected from patients diagnosed with testicular SEM at The First Affiliated Hospital of Nanjing Medical University, the No. 1 People’s Hospital of Nanjing Medical University from 2014 to 2020. The tissue fragments collected after surgical resection were immediately transferred into liquid nitrogen and stored at −80° before use. Every patient had written informed consent. This study was approved by the Ethics Committee of Nanjing Medical University.

### Cell Line and Cell Culture

Human SEM cell line (TCam-2) was used in this study. The TCam-2 cell line was purchased from Huatuo Bioscience Technology Limited Company (Shenzhen, China). Cells were cultured in DMEM medium + 10% fetal bovine serum along with penicillin (100 U/ml) and streptomycin (100 μg/ml, Invitrogen, Carlsbad, CA) at 37°C with 5% CO_2_. Although Eppelmann et al. demonstrated that the TCam-2 cell line was heterogenous, the cell line is still considered as an optimum seminoma cell model and extensively used in relevant studies (18–20).

### Cell Transfection

TCam-2 cells were transfected with constructed lentiviral vectors by GenePharma Gene Company (Shanghai, China) to enhance or silence the expression miR-483-3p. Cells transfected with the corresponding empty lentiviral vector were taken as the negative control group. TCam-2 cells were infected with the lentiviruses based on the protocols and used polybrene (Hanbio, Shanghai, China) as the transfection reagents. The cell culture mediums were replaced to fresh after 24 h. After cell growth for 24 h, 5 μg/ml puromycin (Thermo Fisher Scientific, Shanghai, China) was added into the medium. Finally, positive cells were selected after 2 weeks. In addition, cells were transfected with pcDNA3.1-MMP9 (GenePharma Gene, Shanghai, China), empty pcDNA3.1 plasmid (GenePharma Gene, Shanghai, China) pcDNA3.1-KLF9 (GenePharma Gene, Shanghai, China). Lipofectamine 3000 (Thermo Fisher Scientific, Shanghai, China) was used as cell transfection reagents according to the protocols.

### Reverse Transcription-Quantitative Polymerase Chain Reaction

The total RNAs from the 20 pairs of clinical samples and cultured TCam-2 cells were extracted using TRIzol reagent (Invitrogen, Carlsbad, CA). The cDNA was synthesized using HiScript^®^ II Q RT SuperMix for qPCR (Vazyme Biotech Co., Ltd., Nanjing, China) according to relevant protocols. miR-483-3p, MMP9 and KLF9 mRNA levels were quantified with StepOne Plus Realtime PCR system (Applied Biosystems; Thermo Fisher Scientific, Inc.). U6 and GAPDH were used as an internal standard control for miRNA and mRNA detection, respectively. The relative expression levels of miR-483-3p, MMP9 and KLF9 were calculated using the 2-ΔΔCq method based on the CT values. The following primers were applied to RT-qPCR:

miR-483-3p:

forward: 5′-CGCGTCACTCCTCTCCTCC-3′,

reverse: 5′-AGTGCAGGGTCCGAGGTATT-3′;

MMP9:

forward: TGTACCGCTATGGTTACACTCG,

reverse: GGCAGGGACAGTTGCTTCT;

KLF9:

forward: 5′-CTGGTTGCTGGGACTGTAGC-3′,

reverse: 5′-GTTTTCCAGCTCCCAAACAG-3′;

U6:

forward: 5′-CTCGCTTCGGCAGCACA-3′,

reverse: 5′-AACGCTTCACGAATTTGCGT-3′;

GADPH:

forward: 5′-CCCAGCCTCAAGATCATCAGCAATG-3′,

reverse: 5′-ATGGACTGT GGTCATGAGTCCTT-3′.

### Cell Proliferation Assay

The cell proliferation of TCam-2 cells was measured using the cell counting kit-8 assay (CCK-8) (Dojindo, Kyushu, Japan). Cells were counted and seeded into the 96-well plate with 3,000 cells/well, and then cultured for 24, 48, 72, and 96 h. The absorbance of cultured TCam-2 cells was measured with the micro-plate reader at 450 nm after CCK-8 addition for 2.

### Colony Formation Assay

600 cells per well of TCam-2 cells were seeded into 6-well plates and then cultured in medium containing 10% FBS for 2 weeks. The colonies were fixed with 4% paraformaldehyde for 5–10 min and stained 20 min with 0.1% crystal violet. Finally, the colonies of TCam-2 cells with diameters >2 mm were counted under the microscope, and each group was repeated three times.

### Transwell Cell Migration and Invasion Assay

The 24-well transwell chambers (Corning, NY, USA) with or without the Matrigel (Invitrogen) were used to perform the transwell assays. 1^104^ TCam-2 cells were seeded into the upper chambers in serum-free medium, while the serum-supplied medium was added to the lower chamber in 24-well plates. Afterwards, the cells in the bottom chambers were fixed with methanol stained 20 min with 0.1% crystal violet after incubation at 37°C for 48 h. Cells on the surface of the upper chambers were erased with a cotton swab. Numbers of stained cells in the bottom chambers were assessed from five randomly selected fields, and the data were summarized from three individual experiments.

### Luciferase Report Assay

The pmirGLO Dual-Luciferase miRNA Target Expression Vector is designed to quantitatively evaluate microRNA (miRNA) activity by the insertion of miRNA target sites 3′ of the firefly luciferase gene. The wild type MMP9 3′UTR sequence which contains predicted binding sites of miR-483-3p, and the mutant sequence of MMP9 3′UTR altering certain bases in the binding sites were respectively inserted into the constructed pGL3 promoter vector (Genscript, Nanjing, China). TCam-2 cells were co-transfected with luciferase reporter vectors and miR-483-3p with mimic or negative vector. Eventually, the relative luciferase activities were measured with a Dual Luciferase Assay Kit (Promega, USA) after transfection for 48 h.

### Western Blotting

TCam-2 cells or tissue fragments were collected and lysed in the lysis buffer (Beyotime, Shanghai, China) containing PMSF and protease inhibitor (Beyotime, Shanghai, China) and protease inhibitor (Thermo Fisher Scientific, Shanghai, China) for 30 min on ice, and then centrifuged at 14,000 × g at 4°C for 15 min. The concentrations of extracted protein lysate were calculated with a BCA Protein Assay kit (Beyotime, Shanghai, China) and were then adjusted to consistency. 10% sodium dodecyl sulfate polyacrylamide gel electrophoresis (SDS-PAGE) was used to separate the protein lysate, and then the proteins in the separating gel were transferred onto the polyvinylidene fluoride (PVDF) membrane (Millipore, USA). 5% non-fat dry milk dissolving in TBST was used to block the PVDF membranes for 2 h at room temperature. Afterwards, the membranes were probed with respective primary antibodies (MMP9, KLF9 and GAPDH) overnight at 4°C, and incubated with the HRP-conjugated secondary antibody (ZB-2301, ZSGB-BIO, Beijing, China) for 2 h at room temperature. Anti-GAPDH polyclonal antibody (10494-1-AP, Proteintech, Wuhan, China) was taken as the control. The primary antibodies of KLF9 (ab227920) and MMP9 (ab38898) are obtained from Abcam (Cambridge, UK). The primary antibodies were diluted at 1:1,000 with the primary antibody dilution Buffer (Beyotime), while the secondary antibody was diluted at 1:5,000 with the secondary antibody dilution Buffer (Beyotime).

### *In Vivo* Xenograft Model

The animal experiments in this study were approved by the Animal Research Ethics Committee of Nanjing Medical University. The 5-week-old female BALB/c nude mice were purchased from the animal center of Nanjing University and subsequently randomly divided into two groups consisting of five mice each. TCam-2 cells (2 × 10^7^) of miRNA-483 over-expression group, and the negative control group were injected subcutaneously into the flank of each mouse. Tumor size was calculated and recorded once a week. After 6 weeks, the tumors were removed, photographed, and weighed. Moreover, parts of the tumors were fixed with 4% paraformaldehyde for 24 h, and embedded in paraffin for immunohistochemistry staining.

### Immunohistochemistry

The expression levels of MMP9 were examined by immunohistochemistry staining. Tissues were sliced and deparaffinized. Subsequently, sections were incubated with anti-MMP9 (ab38898, Abcam, Cambridge, UK) antibody overnight at 4°C. Then the sections were washed three times with the phosphate buffered saline. After incubation with goat anti-rabbit IgG for 30 min, all the sections were stained with the 3,3′-diaminobenzidine (DAB).

### Chromatin Immunoprecipitation

The ChIP analysis was conducted with a ChIP assay kit (Millipore, 17-610) based on manufacturer’s instructions. A total of about 4 × 10^7^ Tcam-2 cells were collected, and 1% formaldehyde was used to crosslink the proteins to DNA for 25 min. Chromatin was sheared to fragments with the size of 100–500 bp by sonicating the lysate. After dislodging insoluble substance by centrifugation, 100 μl DNA/protein complexes were taken as input. The samples were incubated with KLF9 antibody and normal rabbit IgG, and the protein A/G beads overnight at 4°C. After incubation at 65°C for 4 h, the crosslinking of input, and the samples were reversed. Afterwards, phenol/chloroform was used to recover DNA from the samples. Finally, promoter binding was evaluated *via* RT-qPCR with relevant primers (forward: 5′-AGGGAGGTGTGAGGTGTCTG-3′, reverse: 5′-ACATACCAGGGTGGGCTTCT-3′) referring to the upstream of miRNA-483-3p and putative binding site.

### Statistical Analysis

The Fisher’s Exact Test was applied to analyze the differences of data shown in **Table 1**. The significant differences of other results were assessed using Student’s unpaired t test. Data in this study were obtained from three independent experiments and were presented as mean ± standard deviation (SD). The software SPSS 22.0 (IBM, Armonk) was utilized to perform all the statistical analyses, and a *P <*0.05 was considered significant.

**Table 1:** Association of miRNA-483-3p expression with clinicopathological characteristics of testicular seminoma patients.

Parameters	No. of cases (%)	miR-483-3p expression		*P*-value
		Low (%)	High (%)	
Age (years)				0.350
≤30	07 (35%)	5	2	
>30	13 (65%)	5	8	
Gender				–
Male	20 (100%)	10	10	
Histology				–
Seminoma	20 (100%)	10	10	
TMN Stage				0.011
I	14	4	10	
II-III	6	6	0	
Survival				0.474
alive	18	8	10	
dead	2	2	0	

## Results

### miR-483-3p Is Down-Regulated in SEM Tissues and Significantly Associated With Tumor Stage

**Figure 1:**
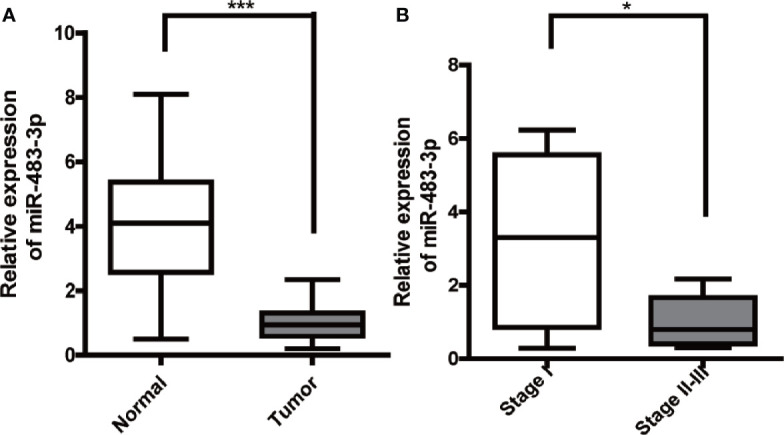
Expression of miR-483-3p in testicular SEM clinical samples. **(A)** The expression levels of miR-483-3p in 20 pairs of human SEM tissues and paracancerous tissues. **(B)** The expression levels of miR-483-3p in SEM patients with different tumor stages. Data are represented as mean ± SD. *p < 0.05, **p < 0.01, ***p < 0.001. SEM, seminoma.

Firstly, we performed RT-qPCR to detect the mRNA level of miR-483-3p in 20 pairs of SEM and paracancerous tissues from patients with testicular SEM. As shown in **Figure 1A**, the expression level of miR-483-3p in SEM tissues was significantly decreased compared with adjacent normal tissues. In addition, miR-483-3p expression was significantly related to TMN stage in SEM patients (*P* = 0.026) in **Table 1**. Meanwhile, we could also see clearly from **Figure 1B** that miR-483-3p was lower-expressed in tumors of stages II–III than in tumors of stage I.

### miR-483-3p Suppresses Proliferation, Invasion, and Migration of SEM Cells

**Figure 2:**
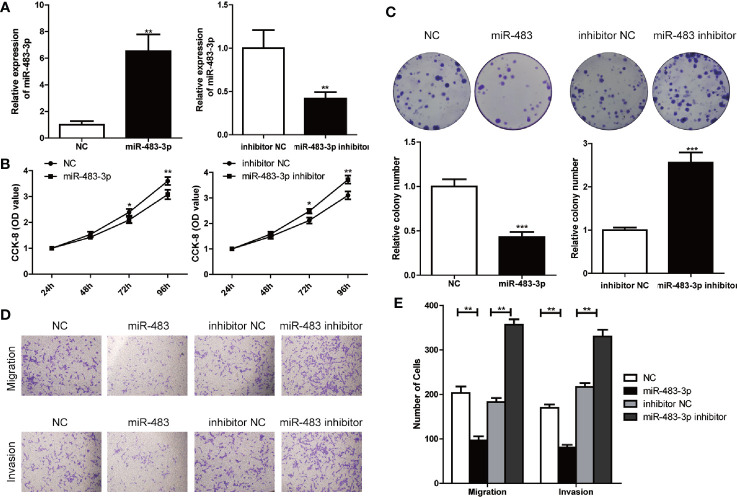
miR-483-3p suppresses proliferation, invasion, and migration of TCam-2 cells. **(A)** The results of miR-483-3p expression in TCam-2 cells transfected with miR-483 mimics and miR-483 inhibitor lentivirus respectively were validated by RT-qPCR; **(B)** CCK-8 was used to determine the proliferation of transfected TCam-2 cells; **(C)** The abilities of cell colony formation was detected in TCam-2 cells after miR-483-3p expression alteration; **(D, E)** Results of transwell and invasion assays for TCam-2 cells transfected with miR-483 mimics or miR-483 inhibitor lentivirus. Data are represented as mean ± SD. *p < 0.05, **p < 0.01, ***p < 0.001.

To further explore the role of miR-483-3p in SEM, we used TCam-2 cell line for subsequent research. TCam-2 cells were transfected with lentiviral constructs to the over-express or inhibit the expression of miR-483-3p, and the transfection efficiencies were validated by RT-qPCR (**Figure 2A**). Results of the CCK-8 assay showed that the inhibition of miR-483-3p significantly promoted the proliferation of TCam-2 cells, while over-expressed miR-483-3p impeded the cell growth (**Figure 2B**). In addition, we also performed the colony formation assay to investigate the effect of miR-483-3p on the cell proliferation. As shown in **Figure 2C**, down-regulated miR-483-3p significantly decreased the cell colony formation efficiency, whereas increased miR-483-3p expression markedly enhanced the ability of colony formation. The above results indicated that miR-483-3p plays a negative role on cell proliferation in SEM.

Subsequently, we performed cell transwell migration and invasion assays to evaluate the potential role of miR-483-3p on the migration and invasion ability of TCam-2 cells. The transwell migration assay revealed that decreased level of miR-483-3p promoted the cell migration, while over-expressed miR-483-3p significantly weakened the ability of migration of TCam-2 cells; and the transwell invasion assays showed similar results (**Figures 2D, E**). These findings suggested the over-expression of miR-483-3p significantly inhibited the migration and invasion ability of SEM cells.

### miR-483-3p Negatively Regulates MMP9 by Directly Targeting the 3′-UTR

**Figure 3:**
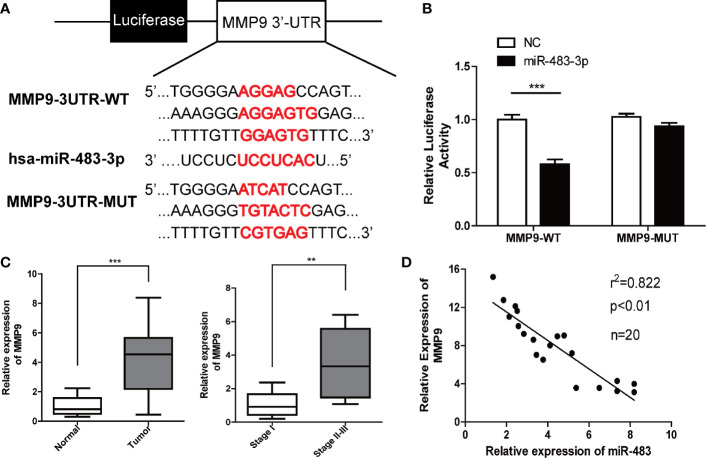
miR-483-3p regulates MMP9 expression by directly binding its 3′UTR. **(A)** Potential binding sites between MMP9 3′UTR and miR-483-3p; **(B)** Luciferase activity was analyzed in cells co-transfected with miR-483 mimics or negative control with pGL3-MMP9-WT or pGL3-MMP9-MUT. **(C)** The relative mRNA expression level of MMP9 in human testicular SEM clinical samples; **(D)** A negative correlation was observed between miR-483-3p and MMP9 in SEM samples. Data are represented as mean ± SD. *p < 0.05, **p < 0.01, ***p < 0.001. SEM, seminoma; MMP9, Matrix metallopeptidase 9.

To further explore the downstream regulatory mechanism of miR-483-3p, we identified MMP9 as a potential target gene for miR-483-3p with the online bioinformatics database TargetScan (http://www.targetscan.org/vert_72/). Base on the potential binding regions in the 3′-UTR of MMP9, we designed luciferase reporter plasmids containing the predicted binding sties in the 3′-UTR of MMP9 (MMP9-WT) or mutated MMP9 (harboring deletions of the putative targeting sites, MMP9-MUT) (**Figure 3A**). As shown in **Figure 3B**, the luciferase activity was significantly decreased when co-transfected with the MMP9-WT and miR-483-3p mimics, whereas was not affected when co-transfected with the MMP9-MUT. These results revealed that MMP9 might be a direct functional target gene of miR-483-3p.

Furthermore, we validated that the expression of MMP9 by RT-qPCR in the 20 pairs of tumor and paracarcinoma tissues. As shown in **Figure 3C**, the expression of MMP9 was upregulated in SEM tissues. Besides, the tumor tissues of the SEM patients with stages II–III shows a higher level of MMP9 than those SEM patients with stage I. What’s more, a negative correlation was found between miR-483-3p and MMP9 expression (**Figure 3D**).

### Over-Expression of miR-483-3p Impedes Xenograft Tumor Formation *In Vivo*

**Figure 4:**
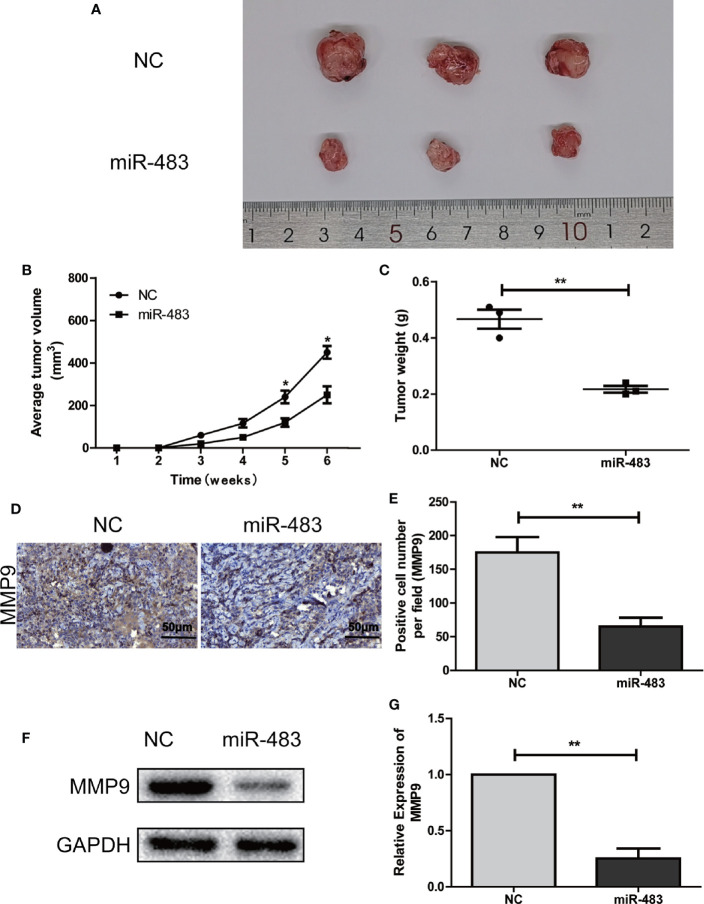
miR-483-3p inhibits SEM growth *in vivo*. **(A–C)** The tumor formation in the control group and the miR-483 group; **(D, E)** Immunohistochemical staining was used to detect the expression of MMP9 in subcutaneous xenograft tumors of the control group or the miR-483 group; **(F, G)** Western Bolt was used to detect the expression of MMP9 in subcutaneous xenograft tumors of the control group or the miR-483 group. Data are represented as mean ± SD. *p < 0.05, **p < 0.01, ***p < 0.001. SEM, seminoma; MMP9, Matrix metallopeptidase 9.

To validate whether miR-483-3p inhibits the SEM growth *in vivo*, we performed subcutaneous xenograft tumor models using the transfected TCam-2 cells. As shown in **Figures 4A–C**, the mice inoculated with TCam-2 cells of over-expressed miR-483-3p could reduce tumor volumes and tumor weights than the mice in the negative control group. In addition, the IHC and Western blotting assays revealed that the level of MMP9 in miR-483-3p over-expression group was obviously lower than that in the negative control group (**Figures 4D–G**). In general, these results demonstrated that the over-expression of miR-483-3p impeded the tumor growth *in vivo*.

### miR-483-3p Suppresses Proliferation, Invasion and Migration of SEM Cells by Targeting MMP9

**Figure 5:**
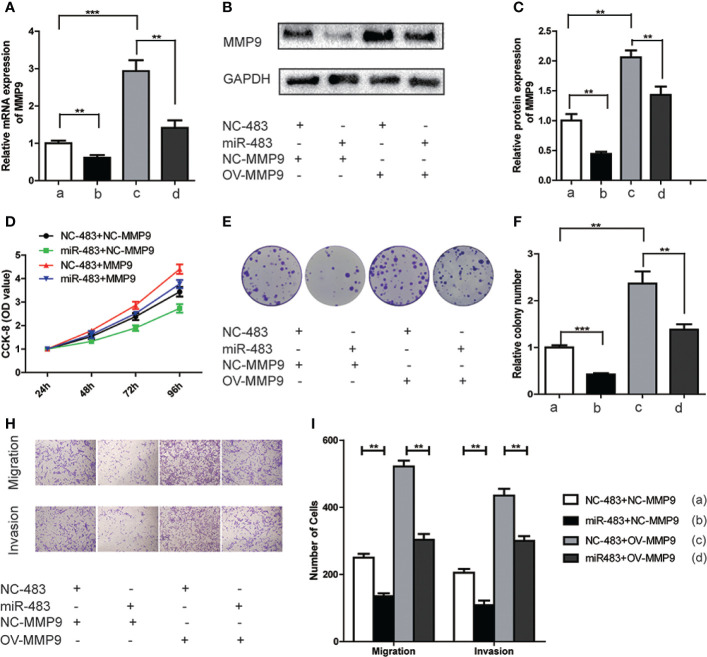
miR-483-3p suppresses proliferation, migration, and invasion in TCam-2 cells by targeting MMP9. TCam-2 cells were co-transfected with pcDNA3.1-MMP9, Lv-miR-483, and respective control vectors. **(A)** The mRNA expression of MMP9 was verified by RT-qPCR in co-transfected TCam-2 cells; **(B, C)** The protein expression of MMP9 was verified by Western Bolt in co-transfected TCam-2 cells; **(D)** The results of CCK-8 in co-transfected TCam-2 cells; **(E)**. **(F)** The abilities of colony formation in co-transfected TCam-2 cells; **(G, H)** The results of migration and invasion assays in co-transfected TCam-2 cells. Data are represented as mean ± SD. *p < 0.05, **p < 0.01, ***p < 0.001. MMP9, Matrix metallopeptidase 9; (a) NC-483+NC-MMP9; (b) miR-483+NC-MMP9; (c) NC-483+OV-MMP9; (d) miR-483+OV-MMP9.

To further elucidate whether miR-483-3p mediated malignant biological properties by regulating MMP9, we conducted relevant rescue assays. TCam-2 cells (control group and miR-483-3p over-expressed group) were transfected with pcDNA3.1-MMP9 and negative vector respectively. As shown in **Figures 5A–C**, the mRNA and protein levels of MMP9 were significantly decreased in miR-483 mimics group while elevated in MMP9 over-expression (OV-MMP9) group. However, the level of MMP9 expression was rescued in miR-483 + OV-MMP9 group. In addition, the over-expression of MMP9 significantly increased cell growth and colony formation ability, which could be rescued by over-expressed miR-483-3p in TCam-2 cells (**Figures 5D–F**).

Furthermore, over-expressed MMP9 in TCam-2 cells significantly increased the abilities of cell migration and invasion. However, such effect was reversed when simultaneously over-expressed miR-483-3p and MMP9 (**Figures 5G, H**). Overall, the above findings revealed that miR-483-3p suppressed cell proliferation, migration, and invasion by directly targeting MMP9 in SEM cells.

### KLF9 Is an Upstream Regulator of miR-483-3p and Functions as a Tumor Suppressor

**Figure 6:**
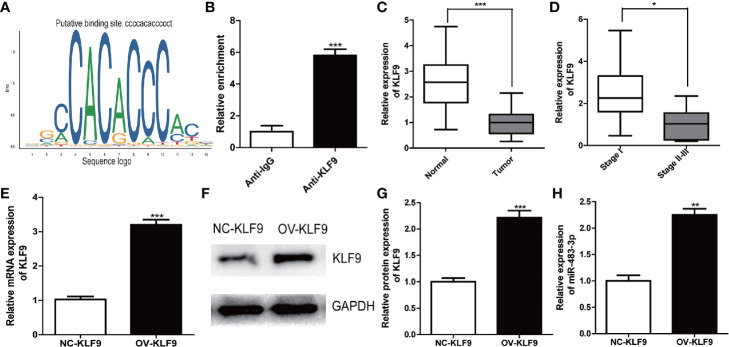
KLF9 was a potential upstream regulator of miR-483-3p. **(A)** Based on JASPAR database, KLF9 might binding to the promoter of miR-483-3p; **(B)** ChIP assay indicated the KLF9 binds to the putative binding site upstream of miR-483-3p; **(C, D)** The mRNA expression of KLF9 in clinical SEM samples; **(E–G)** The TCam-2 cells were transfected with pcDNA3.1-KLF9. RT-qPCR and Western Blot were used to validate the mRNA and protein expression of KLF9; **(H)** The expression of miR-483-3p was detected in TCam-2 cells when KLF9 was over-expressed. Data are represented as mean ± SD. *p < 0.05, **p < 0.01, ***p < 0.001. SEM, seminoma; KLF9, Kruppel like factor 9.

To search for upstream regulators of miR-483-3p in SEM, we identified KLF9 as a potential upstream TF based on JASPAR database by predicting the binding sites of TF with the promoter of miR-483-3p (**Figure 6A**). Furthermore, ChIP assay indicated that KLF9 binds to the putative binding site upstream of miR-483-3p (**Figure 6B**). Subsequently, we validated the differential expression between SEM and paracarcinoma tissues. As shown in **Figure 6C**, KLF9 was down-regulated in SEM tissues when compared to paracarcinoma tissues. Moreover, KLF9 was at a lower level in the SEM patients with stages II–III, compared these with stage I (**Figure 6D**). Therefore, we considered KLF9 as a positive regulator of miRNA-483-3p. The mRNA and protein level of miR-483-3p were significantly increased when KLF9 was over-expressed in TCam-2 cells (**Figures 6E–H**).

**Figure 7:**
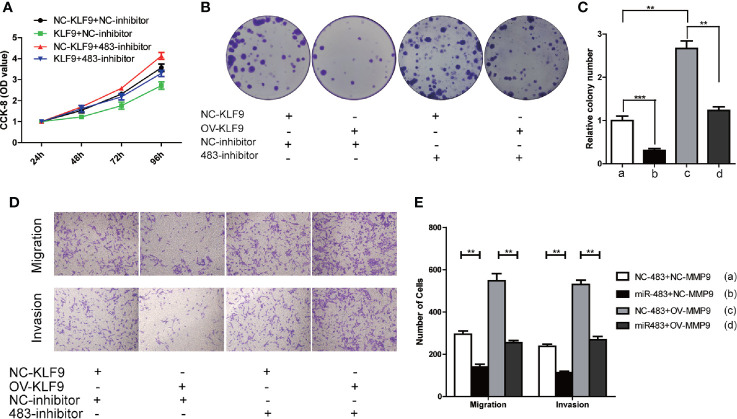
KLF9 functions as tumor suppressor in testicular SEM by regulating miR-483-3p. TCam-2 cells were co-transfected with pcDNA3.1-KLF9, miR-483 inhibitor and respective control vectors. **(A)** The results of CCK-8 in co-transfected TCam-2 cells; **(B, C)** The abilities of colony formation in co-transfected TCam-2 cells; **(D, E)** The results of migration and invasion assays in co-transfected TCam-2 cells. Data are represented as mean ± SD. *p < 0.05, **p < 0.01, ***p < 0.001. SEM, seminoma; KLF9, Kruppel like factor 9.

Subsequently, CCK-8 and cell colony formation assays revealed that KLF9 was a tumor suppressor which could inhibit the growth of TCam-2 cells. However, such inhibiting effect could be reversed when the level of miR-483-3p was decreased (**Figures 7A–C**). Furthermore, increased KLF9 level significantly weakened the abilities of migration and invasion of TCam-2 cells, which could also be rescued by inhibiting miR-483-3p expression (**Figures 7D–E**). Overall, KLF9 might function as a tumor suppressor *via* positively regulating the expression of miR-483-3p.

## Discussion

SEM is the most frequent testicular tumor and always occurs in young men between 20 and 34 years of age (1). Unlike NSEMs, some biomarkers like AFP, b-hCG, LDH are not sensitive for the diagnosis of SEM (21). However, several miRNAs which are not only aberrantly expressed in testicular malignant tissues but also secreted and measurable in the serum, have emerged as promising biomarkers for the diagnosis and monitoring of SEM (22–24). For example, miR-371a-3p, which has been found secreted in approximately 85% of SEM, has specifically exhibited great accuracy in SEM diagnosis (25, 26). Moreover, a lot of miRNAs have been identified as tumor promoting or inhibiting factors in SEM. Liu et al. demonstrated that miR-223-3p could promote the proliferation and inhibit the apoptosis of SEM cells (13). Therefore, exploring the role of different miRNAs in SEM is valuable to understand the mechanisms of SEM occurrence or progression, and will be helpful for the discovery of new biomarkers.

In the recent years, several researches have studied the role of miR-483-3p in the development and progression of several cancers including breast cancer, NSCLC, and colorectal cancer (16, 17, 27). However, the functional role of miR-483-3p in SEM remains unclear. In this study, a series of *in vitro and in vivo* experiments were conducted to clarify the role of miR-483-3p in SEM progression. Firstly, miR-483-3p was found down-regulated in SEM tissues and at a lower level in the patients with stages II–III than those with stage I. Subsequently, TCam-2 cells, the human testicular SEM cell line, were used to investigate the effect of miR-483-3p on cell proliferation, migration and invasion. As expected, the over-expression of miR-483-3p in TCam-2 cells inhibited the abilities of proliferation, invasion, and migration. In addition, *in vivo* experiments suggested that the over-expression of miR-483-3p could also inhibit the growth of SEM. Overall, the results revealed that miR-483-3p was a tumor suppressor in the progression of SEM.

As is well-known, most of the miRNAs function as post-transcriptional regulators of the gene expression through binding to the 3′-untranslated region (3′-UTR) of target mRNAs. Thus, we identified MMP9 as the downstream target gene of miR-483-3p by predicting corresponding binding sites. What’s more, further luciferase reporter assay confirmed such regulatory relation. MMP9 is a well-known tumor promotor and mainly enhances the invasion and metastasis of various tumors (28–30). Moreover, MMP9 also participates in regulating tumor growth (31). Cancer-cell proliferation is significantly suppressed in tumors from MMP9-deficient mice (32, 33), and it has been demonstrated that MMP9 regulates the insulin-like growth factor-triggered to induce cell proliferation in prostate cancer (34). We found that MMP9 was significantly upregulated in testicular SEM tissues and associated with tumor stage. Thus, there was an inverse correlation between miR-483-3p and MMP9 expression in tumor tissues. After enhancing the expression of MMP9, the abilities of cell growth, colony formation, migration, and invasion of TCam-2 cells were all improved. Furthermore, a series of rescue assays revealed that miR-483-3p could reverse the promoting effect of MMP9 on the proliferation, migration and invasion of SEM cells. Based on the results, we proved that miR-483-3p functions antineoplastic effect by decreasing the level of MMP9.

To explore further the mechanism of miR-483-3p in SEM, we speculated that some upstream regulators like TFs might contribute to the dysregulated expression of miR-483-3p. Based on the speculation, we identified KLF9 as a potential upstream regulator of miR-483-3p from JASPAR database. KLF9 has been reported to be associated with many types of solid malignancies (35–37). For example, Li et al. demonstrated that KLF9 suppressed gastric cancer cell invasion and metastasis through inhibiting the level of MMP28 (38). In the present study, we found that KFL9 was down-regulated in SEM tissues, compared to paracarcinoma tissues. When the level of KLF9 was elevated in TCam-2 cells, the expression of miR-483-3p also was increased. In addition, the over-expression of KLF9 led to decreased abilities of cell proliferation, migration and invasion, whereas, the inhibition of miR-483-3p could rescue such effect on TCam-2 cells when KLF9 was over-expressed to some extent. Thus, KLF9 might contribute to the inhibition of malignant behaviors in SEM by modulating miR-483-3p transcription. Therefore, our study has validated that miR-483-3p, mediated by KLF9, is a tumor suppressor in testicular SEM. MiR-483-3p can inhibit cell proliferation, migration, and invasion by targeting MMP9, and is expected to be the monitoring biomarker and the therapeutic target for the clinical treatment of SEM.

## Conclusion

In general, our study suggested that miR-483-3p could inhibit the cell growth, migration and invasion of testicular SEM by targeting MMP9. Moreover, KLF9 is an upstream positive regulator of miR-483-3p and also functions as a tumor suppressor in SEM.

## Data Availability Statement

The raw data supporting the conclusions of this article will be made available by the authors, without undue reservation.

## Ethics Statement

The studies involving human participants were reviewed and approved by the Ethics Committee of Nanjing Medical University. The patients/participants provided their written informed consent to participate in this study. The animal study was reviewed and approved by the Animal Research Ethics Committee of Nanjing Medical University.

## Author Contributions

LFZ, LiZ, WZ: Protocol/project development and funding support; YR, WW, KX: Data collection or management; LeZ, XG, ZQQ: *In vivo* experiments; LeZ, XG, SYL, KZ: In vitro cellular experiments; LeZ, XG, ZQQ: Manuscript writing/editing; SLG, XKS: Manuscript revision and supplementary Experiment. All authors contributed to the article and approved the submitted version.

## Funding

This work was supported by National Natural Science Foundation of China (No. 81670608, 81600514, 81902565), Young Scientists Foundation of Changzhou No.2 People’s Hospital (2019K008), Changzhou Sci & Tech program (CJ20190100), Young Talent Development Plan of Changzhou Health Commission (No. CZQM2020065), Funded by School-level Discipline (No. YJXK202013), Innovation Team (No. XK201803) and Top Talent Project of Changzhou (No. RC201620).

## Conflict of Interest

The authors declare that the research was conducted in the absence of any commercial or financial relationships that could be construed as a potential conflict of interest.

## Supplementary Material

Supplementary Table 1 The promoters of miR-483-3p.
