# Supplementary material for: miR-483-3p, Mediated by KLF9, Functions as Tumor Suppressor in Testicular Seminoma via Targeting MMP9
Source: Front Oncol. 2021 Feb 15;10:596574. doi: 10.3389/fonc.2020.596574 (PMC7917253; doi:10.3389/fonc.2020.596574)
Supplement: Supplementary Table 1 — The promoters of miR-483-3p. [file Table_1.pdf]

cccgctgttcggtttgcgacacgcagcagggaggtgggcggcagcgtcgc
cggcttccaggtaagcggcgtgtgcgggccgggccggggccggggctggg
gcggcgcgggcttgcgccggacgcccggcccttcctccgcccgctcccgg
cccggggcctgcggggctcggcggggcggctgagcccgggggggaggagg
aggaggaggaggaggacggacggctgcgggtcccgttccctgcgcggagc
cccgcgctcaccctggcggcggagctgggggtggggtgggggcgtcggga
agggccgagggaggtgtgaggtgtctgcaggggcgacttcccggtcggtc
tgtgggtgcagggggtgccgcctcacatgtgtgattcgtgccttgcgggc
cctggcctccggggtgctgggtaacgaggaggggcgcggagccgcagaag
cccaccctggtatgttgacgcggtgccagcgagaccgcgagaggaagacg
ggggtgggcggggccaggatggagaggggccgagttggcaggagtcatgg
cagacgccacattcgcgacatctcccccacacccccctctggctctgtccg
caacatttccaaacaggagtcccgggagagggggagaggggctgctggtc
tgaggctaagaagggcagagccttcgacccggagagaggccgcggcccct
gcccagtgggcagcgtggaagtttccatacaaggaggtgggaaggagacc
cccccccccttcactgccctgtgcagagatgagccgggggtgcaggatgg
gagcccatggcacttcgctacgggatggtccagggctcccggttgggggt
gcaggagagaagagactggctgggaggagggagagggcgggagcaaaggc
gcgggggagtggtcagcagggagaggggtgggggggtagggtggagcccgg
gctgggaggagtcggctcacacataaaagctgaggcactgaccagcctgc
